# Supplementary material for: Comprehensive analysis of prime editing outcomes in human embryonic stem cells
Source: Nucleic Acids Res. 2022 Jan 8;50(2):1187–97. doi: 10.1093/nar/gkab1295 (PMC8789035; doi:10.1093/nar/gkab1295)

Supplementary Data

Comprehensive analysis of prime editing outcomes in human embryonic stem cells

Table of Contents

Supplementary Figure 1. Prime editing system.

Supplementary Figure 2. Characterization of H9-iPE2 cells.

Supplementary Figure 3. Comparison of prime editing efficiencies using pegRNAs with varying PBS lengths in H9-iPE2 and HEK293T cells.

Supplementary Figure 4. Generation and characterization of H9-iCas9.

Supplementary Figure 5. Generation and characterization of H9-iABE and H9-iCBE.

Supplementary Figure 6. In vitro differentiation of PE and BE edited cells

Supplementary Figure 7. Targeting the PiZZ 1024 G>A mutation in patient-derived induced pluripotent stem cells with PE3 system.

Supplementary Figure 1. Prime editing system. **(A)** Schematic of PE and pegRNA. **(B)** The nuclease domain (nCas9) of the PE nicks the PAM-containing strand. The liberated 3' end binds to the PBS and RT synthesizes edited DNA using the RT template of the pegRNA. **(C)** Elongation of the 3' end by RT generates a 3' flap that contains the edited sequence. The 3' flap can then be transformed to a 5' flap through flap equilibration. Resolution of the 5' flap by DNA repair machinery incorporates the intended edit into the DNA. **(D)** PE3 system uses an additional sgRNA targeting the non-edited strand to enhance editing.

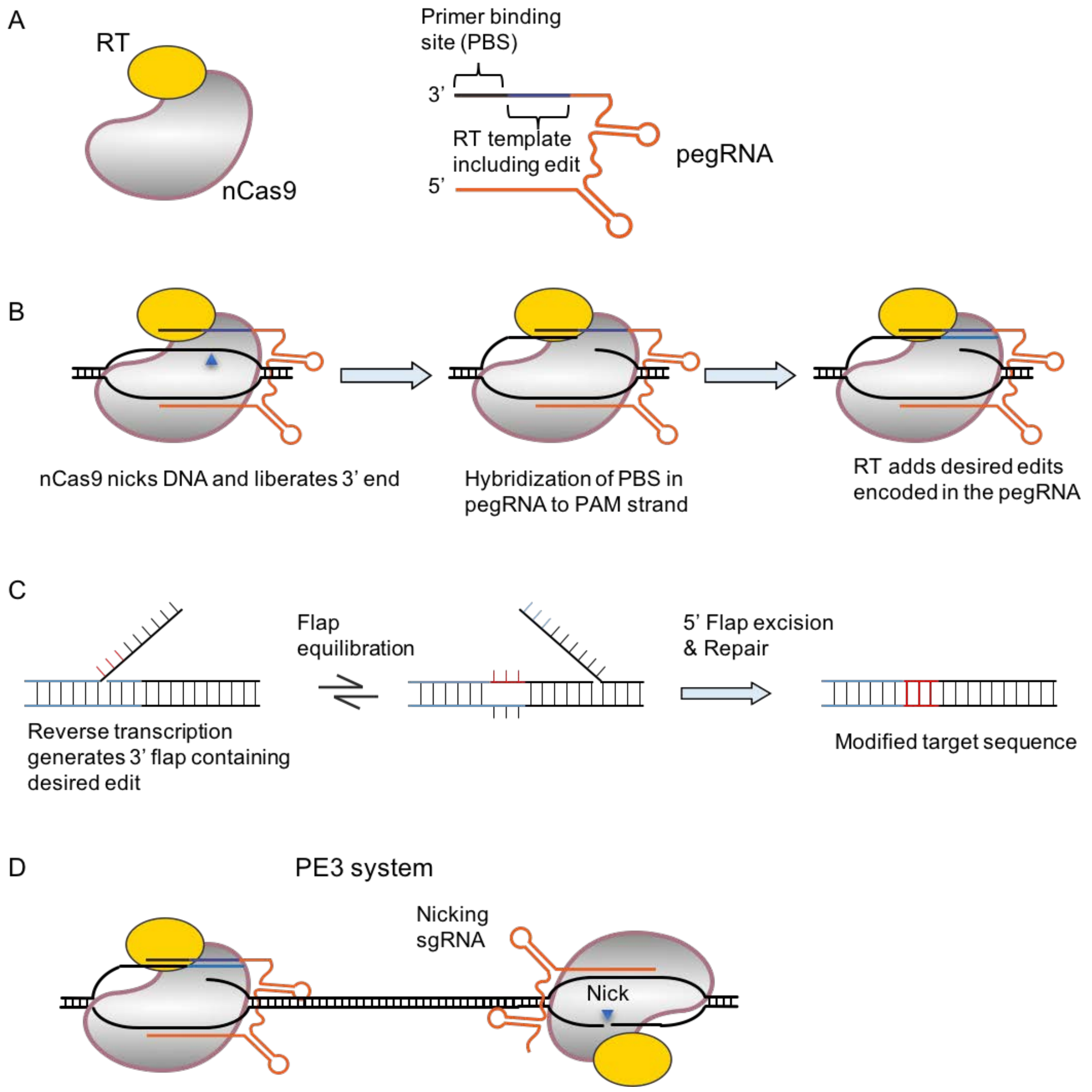

Supplementary Figure 2. Characterization of H9-iPE2 cells. **(A-B)** PCR-based genotyping confirmed homozygous and correct targeted integration of the inducible PE2 expression cassette into the AAVS1 locus. Non-transfected parental cells were used as a negative control for the genotyping. Colored arrows indicate the locations of the primer sets used for genotyping (top panels). The dotted rectangles delineate the areas in the gels shown in Fig. 1 (middle panels). Sanger sequencing was performed to confirm the integration (bottom panels).

A

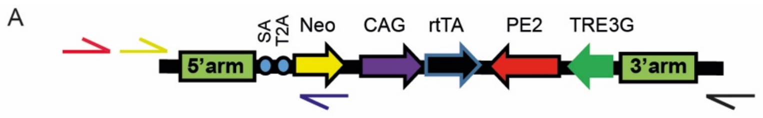

	Line	Forward Primer	Sequence	Reverse Primer	Sequence	DNA	amplicon
AAVS1 locus	2	AAVS1 locus F	CCTGGCCATTGTCACTTTGC	AAVS1 locus R	CCACGTAACCTGAGAAGGGAAT	H9 control	2078
	3	AAVS1 locus F	CCTGGCCATTGTCACTTTGC	AAVS1 locus R	CCACGTAACCTGAGAAGGGAAT	PE2 #5	-
5' transgene junction	4	AAVS1 F2	CTGCCGTCTCTCTCCTGAGT	Neo J R	CTCGTCCTGCAGTTCATTCA	H9 control	-
	5	AAVS1 F2	CTGCCGTCTCTCTCCTGAGT	Neo J R	CTCGTCCTGCAGTTCATTCA	PE2 #5	1258
	6	AAVS1 locus F	CCTGGCCATTGTCACTTTGC	Neo J R	CTCGTCCTGCAGTTCATTCA	H9 control	-
	7	AAVS1 locus F	CCTGGCCATTGTCACTTTGC	Neo J R	CTCGTCCTGCAGTTCATTCA	PE2 #5	1338

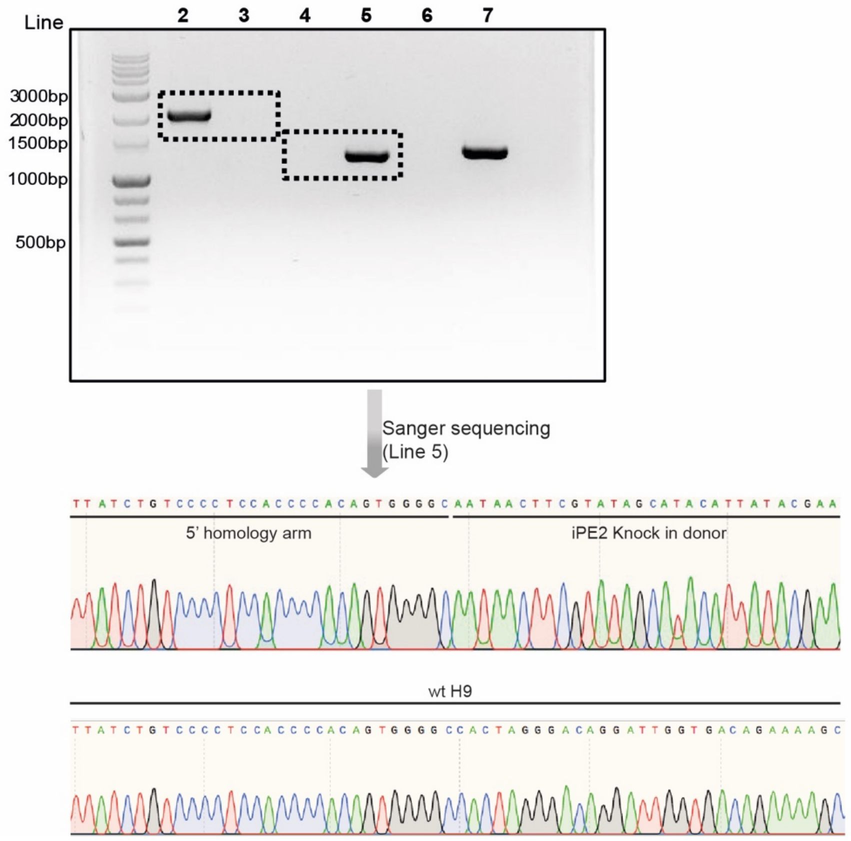

B

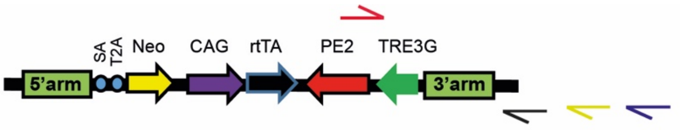

	Line	Forward Primer	Sequence	Reverse Primer	Sequence	DNA	amplicon
3' transgene junction	2	AAVS1 3' J F	GATCTGACGGTTCACTAAACGAGC	AAVS1 locus R	CCACGTAACCTGAGAAGGGAAT	H9 control	-
	3	AAVS1 3' J F	GATCTGACGGTTCACTAAACGAGC	AAVS1 locus R	CCACGTAACCTGAGAAGGGAAT	PE2 #5	1438
	4	AAVS1 3' J F	GATCTGACGGTTCACTAAACGAGC	AAVS1 R2	ACCCAGAGACAGTGACCAAC	H9 control	-
	5	AAVS1 3' J F	GATCTGACGGTTCACTAAACGAGC	AAVS1 R2	ACCCAGAGACAGTGACCAAC	PE2 #5	1578
	6	AAVS1 3' J F	GATCTGACGGTTCACTAAACGAGC	AAVS1 R3	GAATCCCTCCTCTCTGAACC	H9 control	-
	7	AAVS1 3' J F	GATCTGACGGTTCACTAAACGAGC	AAVS1 R3	GAATCCCTCCTCTCTGAACC	PE2 #5	1420

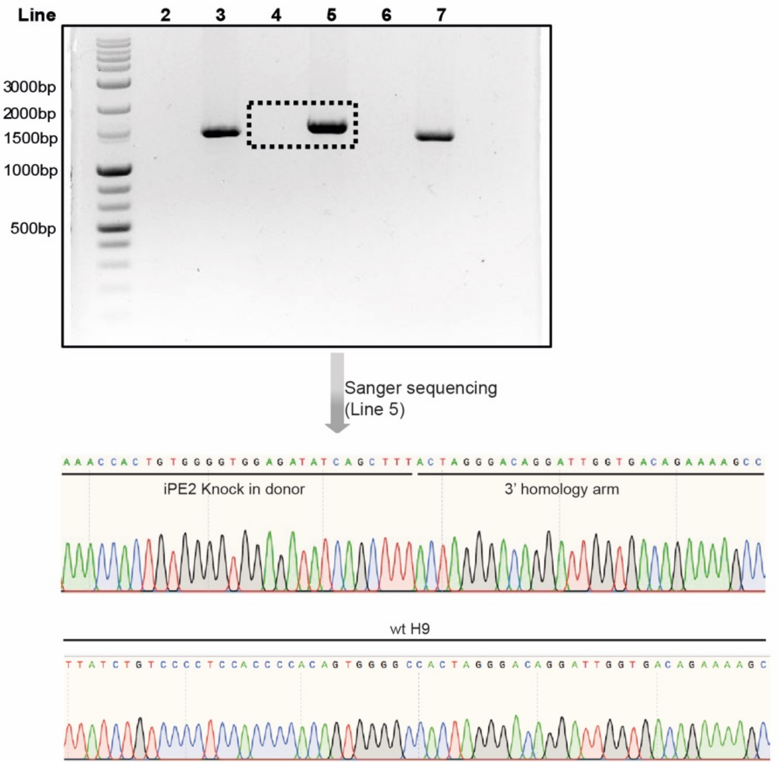

Supplementary Figure 3. Comparison of prime editing efficiencies using pegRNAs with varying PBS lengths in H9-iPE2 and HEK293T cells. **(A)** HEK3 site. **(B)** RNF2 site. Mean ± s.d. of n = 3 independent biological replicates.

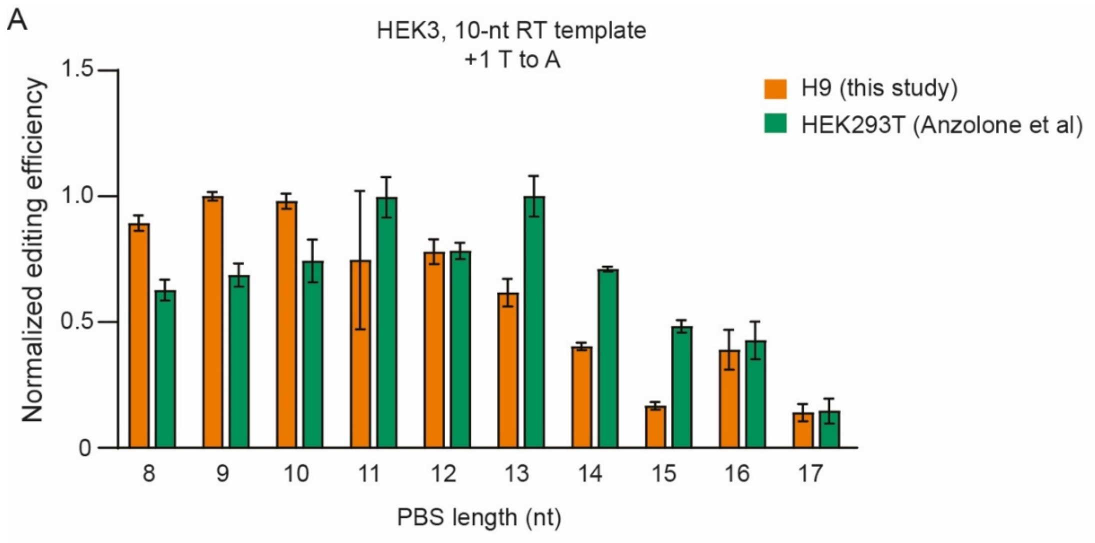

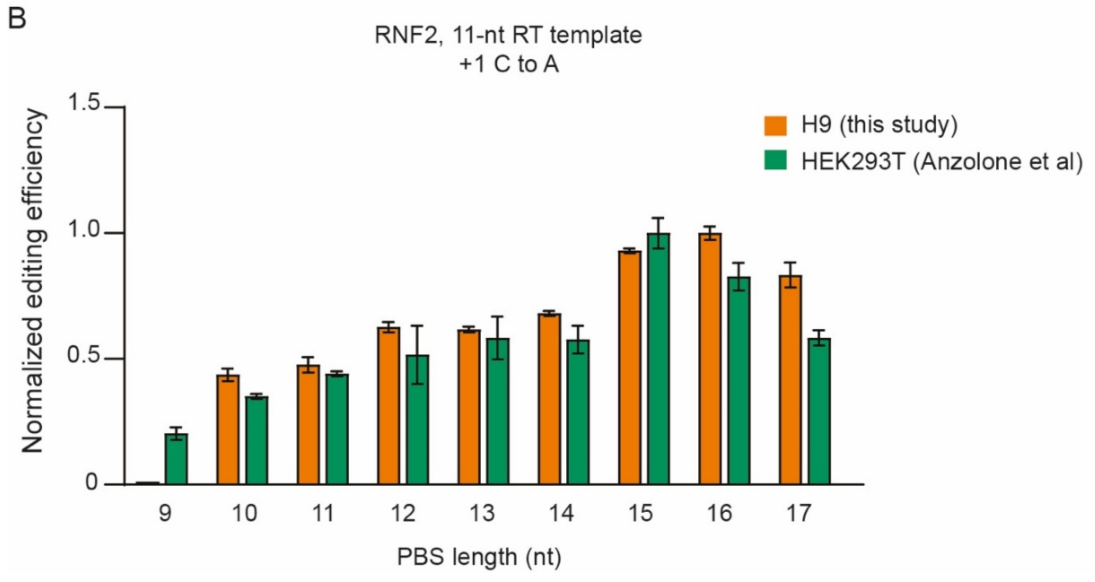

Supplementary Figure 4. Generation and characterization of H9-iCas9. **(A)** Schematic diagram of the strategy for TALEN-mediated targeting of the AAVS1 locus to generate H9-iCas9 cells, in which Cas9 expression is induced by dox. The AAVS1 donor vector contains a cassette in which Cas9 expression is under the control of the dox-inducible TRE3G promoter. SA, splice acceptor; T2A, self-cleaving 2A peptide; Puro, puromycin resistance gene; rtTA, dox-controlled reverse transcriptional activator; CAG, cytomegalovirus early enhancer/chicken β actin promoter. **(B)** Induction of Cas9 expression by the addition of dox. The Cas9 protein was detected by immunostaining using an anti-Cas9 antibody (green). Nuclei were stained with DAPI (blue). **(C-D)** PCR genotyping from an individual clone demonstrating successful knock-in of the inducible Cas9 expression cassette into the AAVS1 locus. Colored arrows indicate the location of the primer sets used for genotyping.

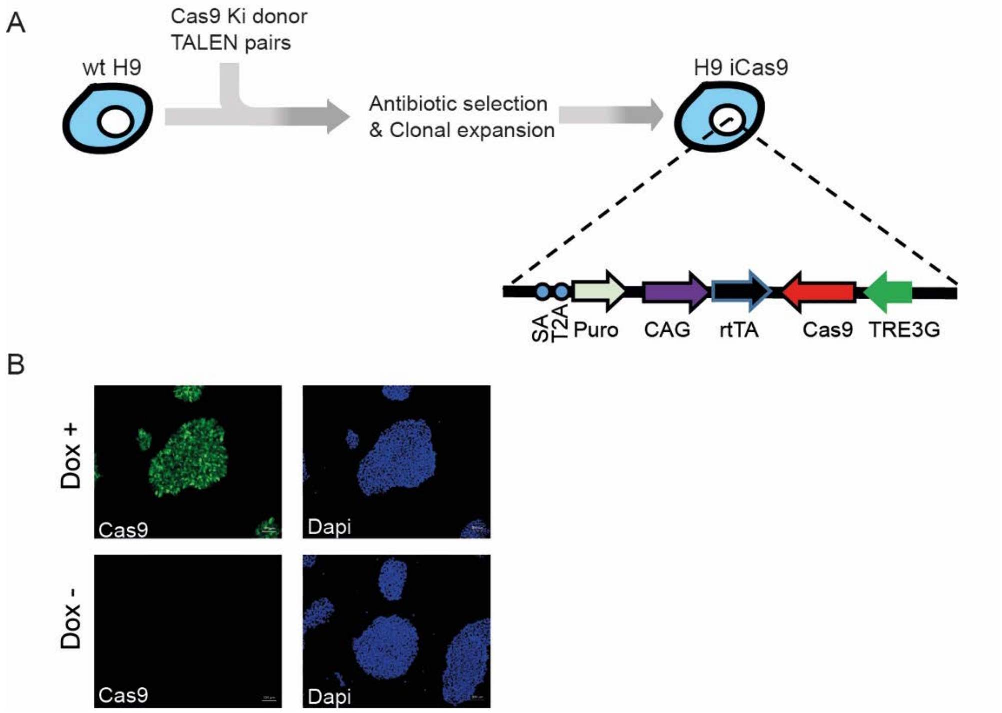

C

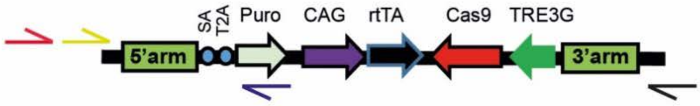

	Line	Forward Primer	Sequence	Reverse Primer	Sequence	DNA	amplicon
AAVS1 locus	2	AAVS1 locus F	CCTGGCCATTGTCACTTTGC	AAVS1 locus R	CCACGTAACCTGAGAAGGGAAT	H9 control	2078
	3	AAVS1 locus F	CCTGGCCATTGTCACTTTGC	AAVS1 locus R	CCACGTAACCTGAGAAGGGAAT	iCas9#1	-
5' transgene junction	4	AAVS1 F2	CTGCCGTCTCTCTCCTGAGT	Puro J R	GTGGGCTTGTACTCGGTCAT	H9 control	-
	5	AAVS1 F2	CTGCCGTCTCTCTCCTGAGT	Puro J R	GTGGGCTTGTACTCGGTCAT	iCas9#1	1068
	6	AAVS1 locus F	CCTGGCCATTGTCACTTTGC	Puro J R	GTGGGCTTGTACTCGGTCAT	H9 control	-
	7	AAVS1 locus F	CCTGGCCATTGTCACTTTGC	Puro J R	GTGGGCTTGTACTCGGTCAT	iCas9#1	1148

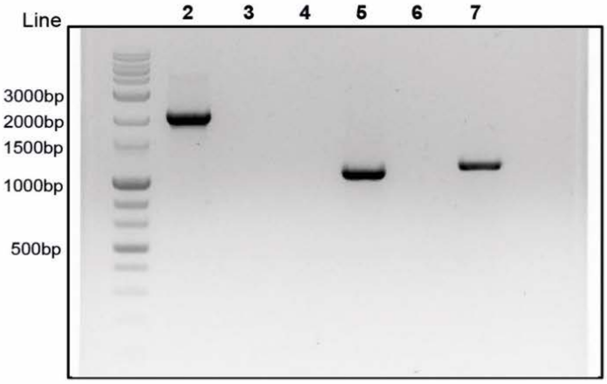

D

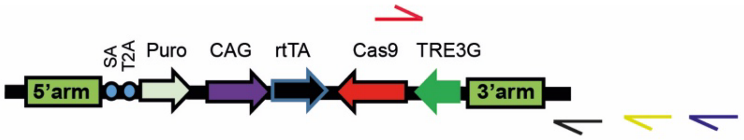

	Line	Forward Primer	Sequence	Reverse Primer	Sequence	DNA	amplicon
3' transgene junction	2	AAVS1 3' J F	GATCTGACGGTTCACTAAACGAGC	AAVS1 locus R	CCACGTAACCTGAGAAGGGAAT	H9 control	-
	3	AAVS1 3' J F	GATCTGACGGTTCACTAAACGAGC	AAVS1 locus R	CCACGTAACCTGAGAAGGGAAT	iCas9#1	1438
	4	AAVS1 3' J F	GATCTGACGGTTCACTAAACGAGC	AAVS1 R2	ACCCAGAGACAGTGACCAAC	H9 control	-
	5	AAVS1 3' J F	GATCTGACGGTTCACTAAACGAGC	AAVS1 R2	ACCCAGAGACAGTGACCAAC	iCas9#1	1578
	6	AAVS1 3' J F	GATCTGACGGTTCACTAAACGAGC	AAVS1 R3	GAATCCCTCCTCTCTGAACC	H9 control	-
	7	AAVS1 3' J F	GATCTGACGGTTCACTAAACGAGC	AAVS1 R3	GAATCCCTCCTCTCTGAACC	iCas9#1	1420

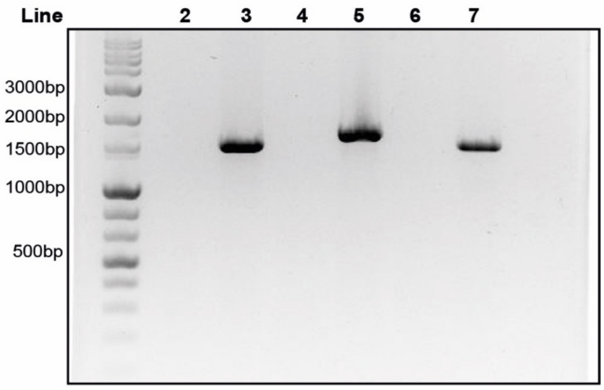

Supplementary Figure 5. Generation and characterization of H9-iABE and H9-iCBE. **(A)** Schematic diagram of the strategy for TALEN-mediated targeting of the AAVS1 locus to generate dox inducible base editor expressing H9 cells. IRES2 EGFP was introduced down stream of AncBE4max and T2A mCherry was fused with ABE8e to track transgene expression. SA, splice acceptor; T2A, self-cleaving 2A peptide; Puro, puromycin resistance gene; rtTA, dox-controlled reverse transcriptional activator; CAG, cytomegalovirus early enhancer/chicken β actin promoter; IRES2, internal ribosome entry site; mCherry, red fluorescent protein; EGFP, enhanced green fluorescent protein. **(B)** Induction of ABE and CBE expression by the addition of dox. **(C-D)** PCR genotyping from an individual clone demonstrating successful knock-in of the inducible ABE and CBE expression cassettes into the AAVS1 locus. Colored arrows indicate the location of the primer sets used for genotyping. **(E)** Representative image of western blot measuring PE2, ABE and CBE protein levels in H9-iPE2, H9-iABE and H9-iCBE cell lines. **(F)** Relative PE2, ABE and CBE protein levels. Blot was analyzed by densitometry using Image J Software

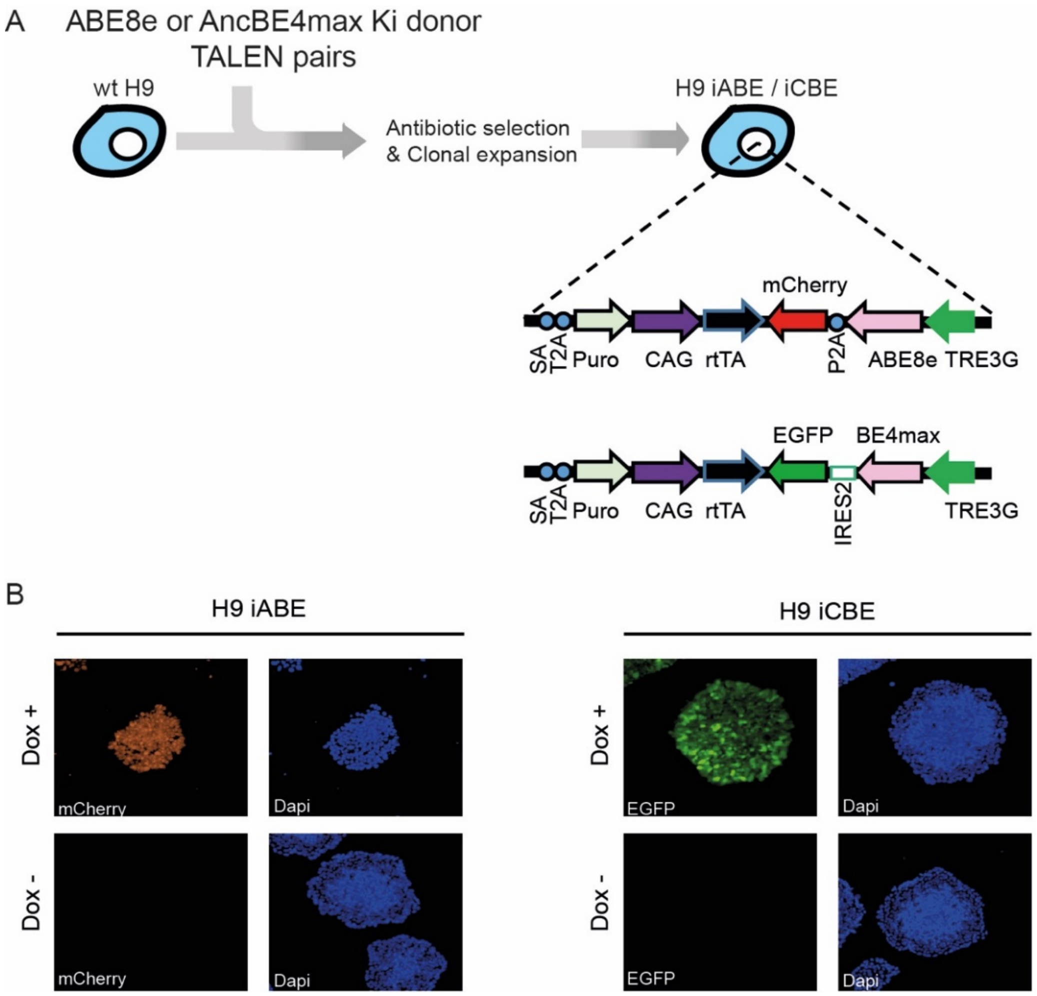

C

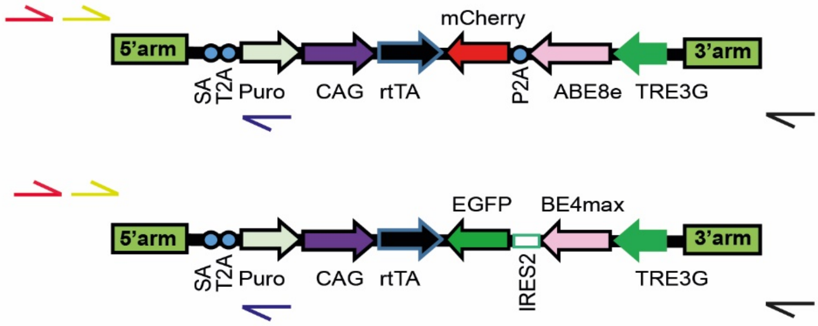

	Line	Forward Primer	Sequence	Reverse Primer	Sequence	DNA	amplicon
AAVS1 locus	2	AAVS1 locus F	CCTGGCCATTGTCACTTTGC	AAVS1 locus R	CCACGTAACCTGAGAAGGGAAT	H9 control	2078
	3	AAVS1 locus F	CCTGGCCATTGTCACTTTGC	AAVS1 locus R	CCACGTAACCTGAGAAGGGAAT	iABE #1<br>iCBE #1	-
5' transgene junction	4	AAVS1 F2	CTGCCGTCTCTCTCCTGAGT	Puro J R	GTGGGCTTGTACTCGGTCAT	H9 control	-
	5	AAVS1 F2	CTGCCGTCTCTCTCCTGAGT	Puro J R	GTGGGCTTGTACTCGGTCAT	iABE #1<br>iCBE #1	1068
	6	AAVS1 locus F	CCTGGCCATTGTCACTTTGC	Puro J R	GTGGGCTTGTACTCGGTCAT	H9 control	-
	7	AAVS1 locus F	CCTGGCCATTGTCACTTTGC	Puro J R	GTGGGCTTGTACTCGGTCAT	iABE #1<br>iCBE #1	1148

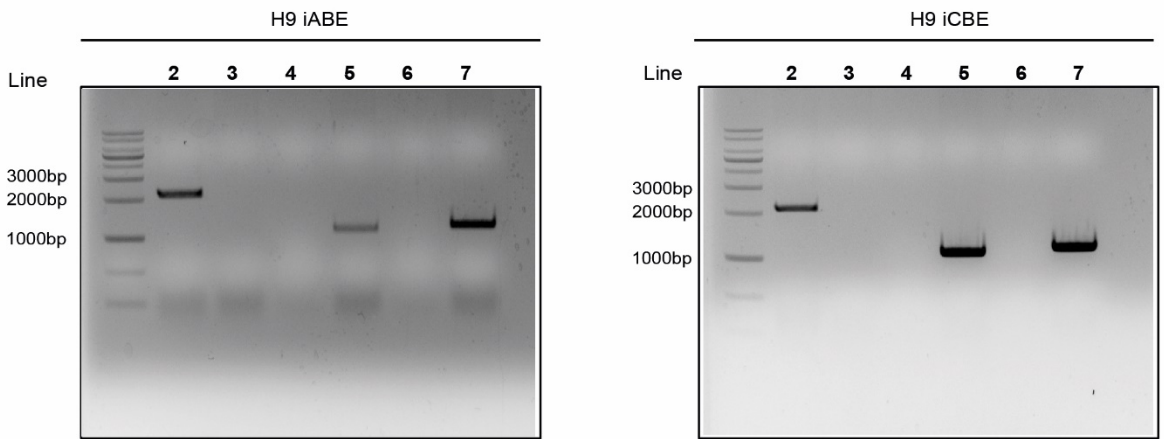

D

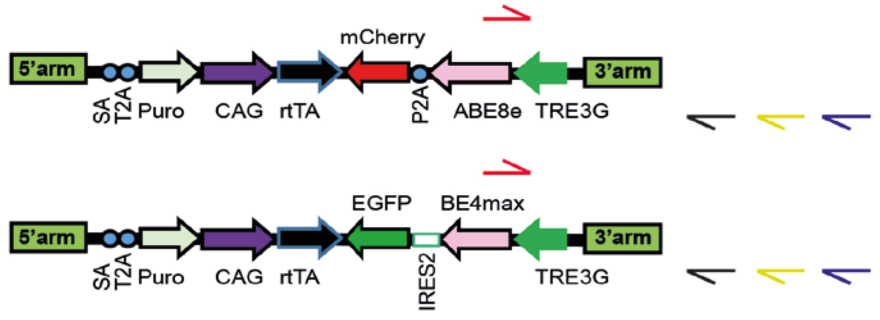

	Line	Forward Primer	Sequence	Reverse Primer	Sequence	DNA	amplicon
3' transgene junction	2	AAVS1 3' J F	GATCTGACGGTTCACTAAACGAGC	AAVS1 locus R	CCACGTAACCTGAGAAGGGAAT	H9 control	-
	3	AAVS1 3' J F	GATCTGACGGTTCACTAAACGAGC	AAVS1 locus R	CCACGTAACCTGAGAAGGGAAT	iABE #1<br>iCBE #1	1438
	4	AAVS1 3' J F	GATCTGACGGTTCACTAAACGAGC	AAVS1 R2	ACCCAGAGACAGTGACCAAC	H9 control	-
	5	AAVS1 3' J F	GATCTGACGGTTCACTAAACGAGC	AAVS1 R2	ACCCAGAGACAGTGACCAAC	iABE #1<br>iCBE #1	1578
	6	AAVS1 3' J F	GATCTGACGGTTCACTAAACGAGC	AAVS1 R3	ACCCAGAGACAGTGACCAAC	H9 control	-
	7	AAVS1 3' J F	GATCTGACGGTTCACTAAACGAGC	AAVS1 R3	ACCCAGAGACAGTGACCAAC	iABE #1<br>iCBE #1	1420

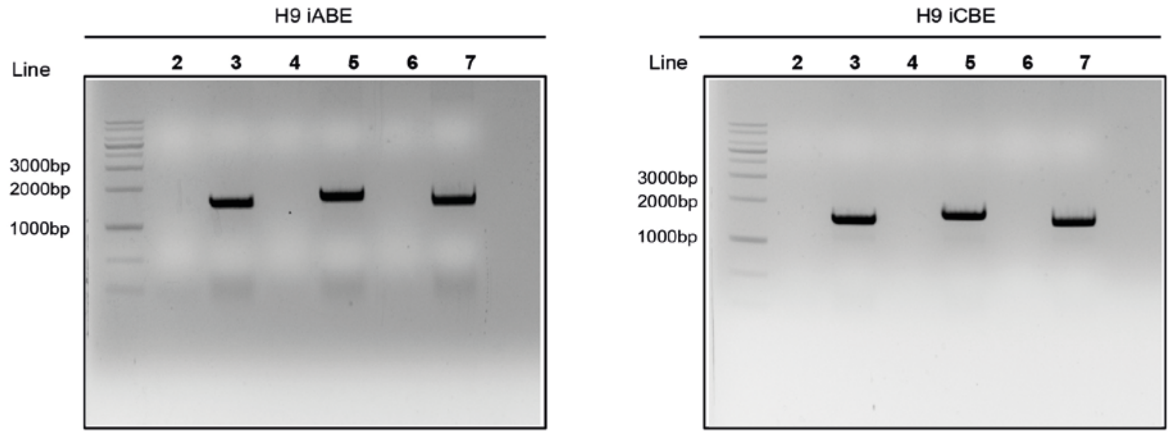

E

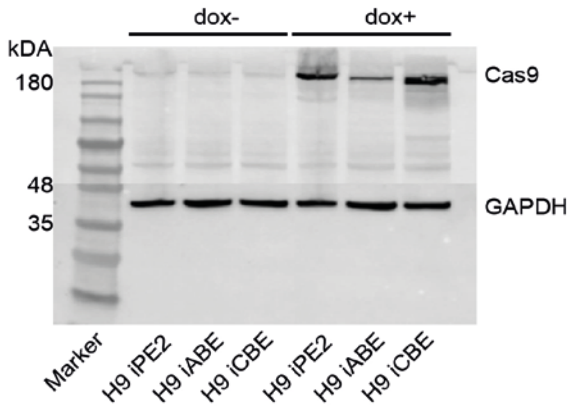

F

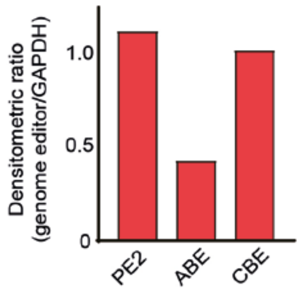

Supplementary Figure 6. In vitro differentiation of PE and BE edited cells. **(A)** Schematic of experiments for in vittro differentiation. enzymes and guide RNAs tested to compare indel frequencies. **(B)** qRT-PCR analysis on the selected lineage genes in the indicated experiment groups.

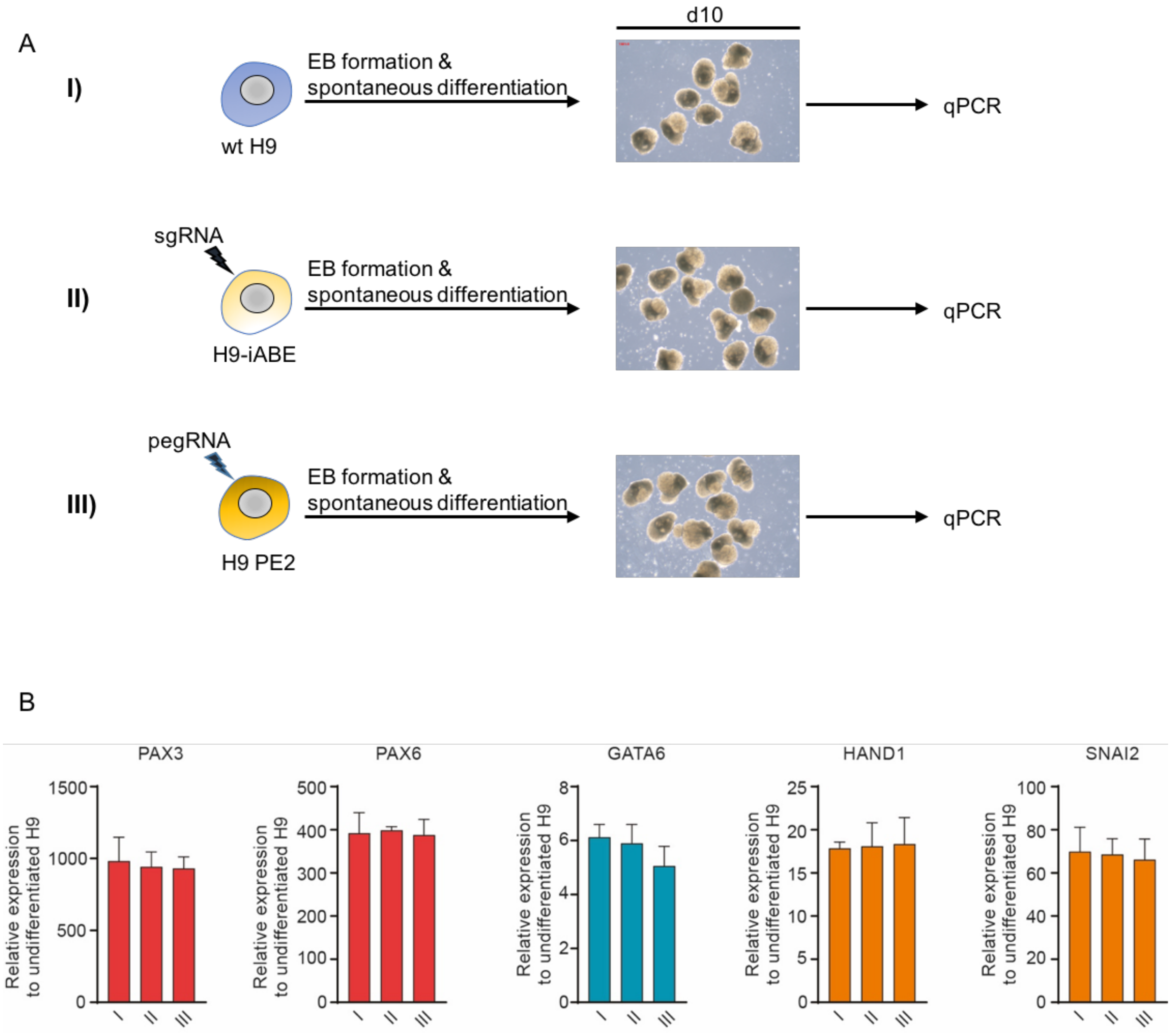

Supplementary Figure 7. Targeting the PiZZ 1024 G>A mutation in patient-derived induced pluripotent stem cells with PE3 system. **(A)** The targeted adenine was at the +24 position (pegRNA1 with NGG PAM) or at the +3 position (pegRNA2 with NG PAM). **(B)** pegRNA1 with varying PBS lengths were tested in the presence of an sgRNA, but the frequencies of the desired edit were at background levels. **(C)** Comparison of indel frequencies mediated by ABE and by PE3 with various RT lengths in pegRNA2.

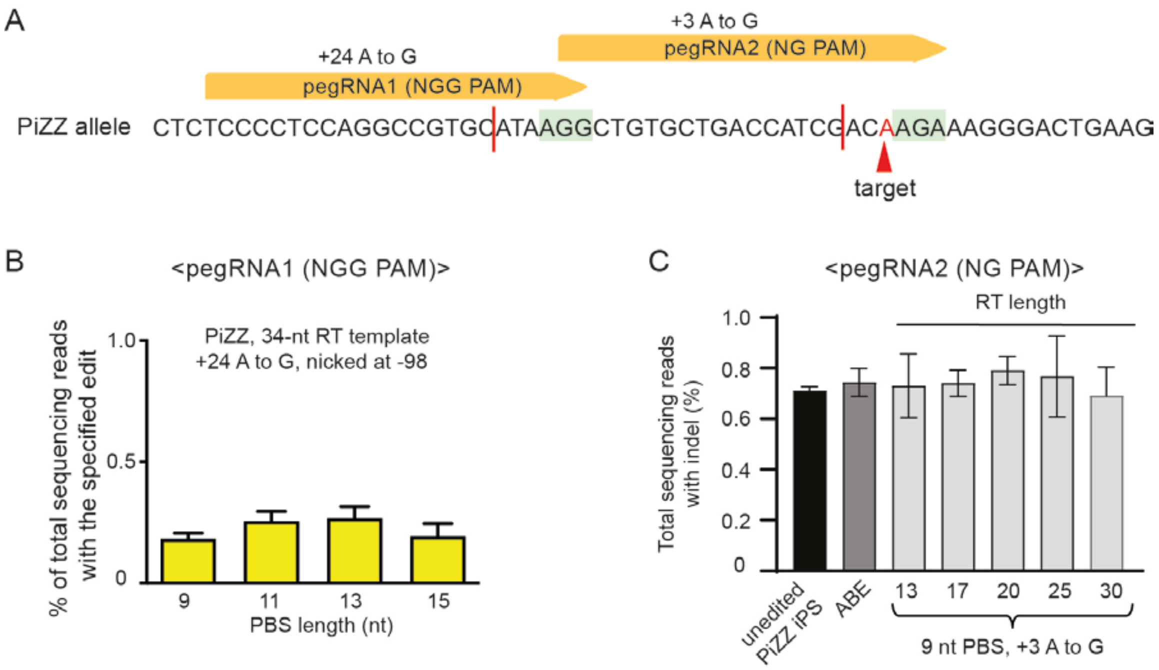

Supplement: gkab1295_Supplemental_Files [file gkab1295_supplemental_files.zip › Supple data_NAR_revision_1214.pdf]
